# Clinicopathological features and prognosis of pseudomyxoma peritonei

**DOI:** 10.3892/etm.2013.1408

**Published:** 2013-11-13

**Authors:** HUAN WANG, XUEJUN WANG, YANFANG JU, JINLIANG WANG, XIN ZHANG, YAO CHENG, JING SUN, YI HU

**Affiliations:** 1 Department of Oncology, The General Hospital of PLA, Beijing 100853, P.R. China; 2 Department of Neurology, Qinghai Women Children’s Hospital, Xining, Qinghai 810000, P.R. China

**Keywords:** pseudomyxoma peritonei, cytoreductive surgery, hyperthermic intraperitoneal chemoperfusion, prognosis

## Abstract

The aim of this study was to evaluate the effects of treatment and the factors influencing the postoperative recurrence and survival time for pseudomyxoma peritonei (PMP). A total of 39 patients with PMP who received treatment were analyzed in The General Hospital of PLA (Beijing, China) between 2002 and 2011. The patients received cytoreductive surgery (CRS) and 25 cases of PMP recurred. Seven patients received postoperative hyperthermic intraperitoneal chemoperfusion (HIPEC). The median follow-up was 40 months. There were eight mortalities in this period. The 5- and 10-year survival rates were 89.0 and 35.0%, respectively. The medians of overall survival (OS) and recurrence time were 37 and 4 months, respectively. Multivariate analyses revealed that pathological subtype was able to influence the recurrence (P=0.042) and OS (P=0.033) times, as an independent prognostic factor. HIPEC was significantly associated with postoperative recurrence time (P=0.017). Patients with disseminated peritoneal adenomucinosis had a more favorable prognosis. CRS combined with HIPEC was able to extend the postoperative recurrence time for patients with PMP.

## Introduction

Pseudomyxoma peritonei (PMP) is a rare disease with an incidence of ~2/10,000 (1). The majority of PMP cases are reported to originate from the ovaries and appendix. Females present more frequently with PMP and the male to female ratio is 1:3.4 (2). The patient usually presents with abdominal pain, bloating, abdominal mass, progressive increase of abdominal circumference, weight loss and fatigue; abdominal percussion dullness and signs of ascites are not obvious. Ascites is not easily removed from the mucinous liquid. This disease shows no marked specificity and misdiagnosis is easy. The course of the disease is long and relapse is common. The present study collected clinical data in The General Hospital of PLA (Beijing, China) between 2002 and 2011. The preoperative evaluation included thoracic and abdominal computed tomography (CT) scans or ultrasound, as well as complete blood tests. Cytoreductive surgery (CRS) and hyperthermic intraperitoneal chemoperfusion (HIPEC) have been the common treatments for PMP since the 1980s. A 5-year survival rate of 86% was reported in a study by Sugarbaker (3). The purpose of the present study was to evaluate the effects of treatment and the prognostic factors influencing the recurrence and overall survival (OS) times for PMP.

## Materials and methods

### Patients

A total of 39 patients were admitted to The General Hospital of PLA between 2002 and 2011. Retrospective analysis of the clinical data of 39 cases of PMP included age, gender, cardinal symptom, image analysis, treatment mode, pathological diagnosis, tumor markers (carcinoembryonic antigen, CEA; alpha-fetoprotein, AFP; cancer antigen 125, CA125) and postoperative adjuvant therapy. The patients met the clinical and pathological diagnostic criteria. The clinicopathological features of the 39 patients with PMP are shown in Table I. Peritoneal lesions were classified into three groups, consisting of disseminated peritoneal adenomucinosis (DPAM), peritoneal mucinous carcinomatosis (PMCA) and peritoneal mucinous carcinomatosis with intermediate or discordant features (PMCA-I/D). The common clinical symptoms and signs were abdominal bulge, abdominal distension, abdominal pain and massive ascites. Four patients were admitted for an abdominal mass. The patients underwent tumor marker measurements. Abdominal ultrasound and CT scans revealed the following: ascites; inward gathering of the intestinal canal; thickening of the peritoneum; widely distributed fluid sonolucent area in the peritoneal cavity, the surrounding of the liver and stomach and the interspaces of the intestines; and thickening and calcification of the peritoneum with widespread and multiple small nodular foci. The informed consent was obtained from the patients or patient’s family.

### Treatment

Surgical resection in combination with perioperative intraperitoneal chemotherapy (PIC) and HIPEC was the major treatment approach for PMP. Intraoperative chemotherapy medication included fluorouracil (5-FU), mitomycin and cisplatin, while HIPEC comprised 5-FU, cisplatin and heating to 43ºC for 60 min. A total of 39 patients with PMP were treated with CRS. HIPEC was performed in seven patients (18%) at a temperature of 43ºC, with cisplatin plus 5-FU. Twenty-two patients received PIC with cisplatin plus 5-FU.

### Statistical analysis

OS and recurrence times were calculated from surgery to the time of mortality or post-surgical disease recurrence. Survival estimates were calculated using the Kaplan-Meier method. The log-rank test was used to assess the significance of the survival distribution. On the basis of univariate analysis, significant variables were included in a Cox proportional hazard model for multivariate analysis. All analyses were performed using SPSS 13.0 statistical software (SPSS, Inc., Chicago, IL, USA). P<0.05 was considered to indicate a statistically significant difference.

## Results

### Clinicopathological features

Among the 39 patients, 15 were male (38%) and 24 were female (62%). The median age was 61 years (range 26–80 years). Thirty-four patients were diagnosed with DPAM (67%), three with PMCA (8%) and two with PMCA-I/D (5%). The baseline marker levels were elevated in 20 patients prior to the surgery (51%), and 15 patients with an elevated marker level demonstrated recurrence following surgery (52%). All patients received surgery and 25 cases recurred. Thirteen patients underwent further surgery. Seven patients received HIPEC following surgery and three cases recurred. The evaluation methods included physical examination, CT scan and blood test.

### Prognosis

**Figure 1:**
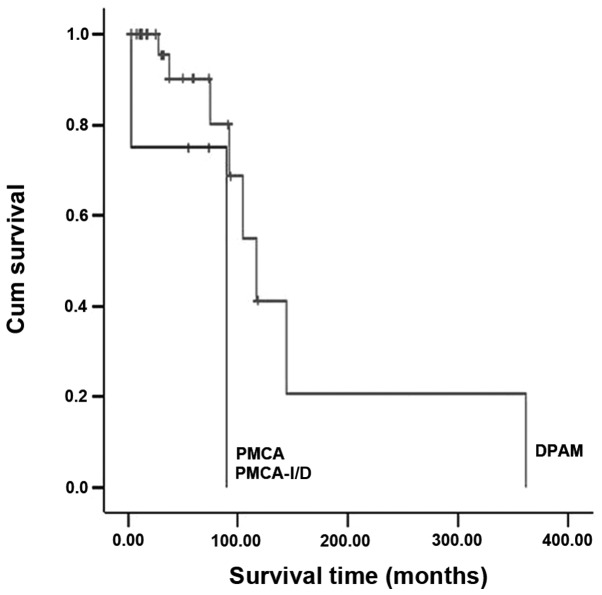
Overall survival according to the pathological type (P=0.048). DPAM, disseminated peritoneal adenomucinosis; PMCA-I/D, peritoneal mucinous carcinomatosis with intermediate or discordant features; PMCA, peritoneal mucinous carcinomatosis.

**Figure 2:**
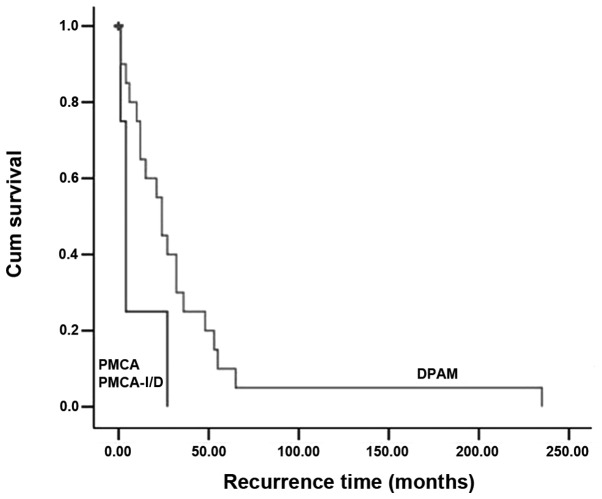
Recurrence-free survival according to the pathological type (P=0.047). DPAM, disseminated peritoneal adenomucinosis; PMCA-I/D, peritoneal mucinous carcinomatosis with intermediate or discordant features; PMCA, peritoneal mucinous carcinomatosis.

**Figure 3:**
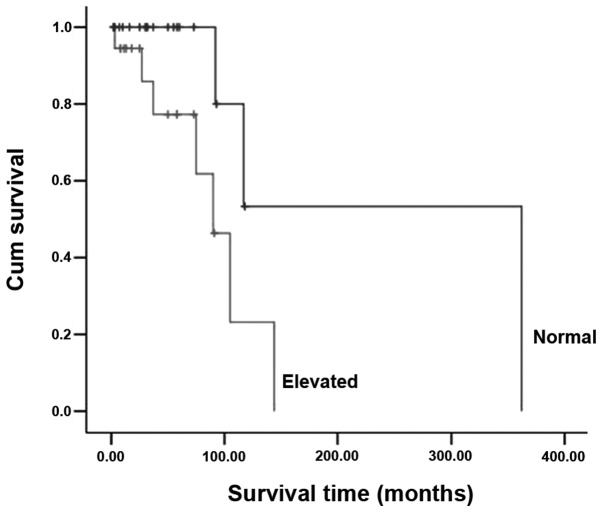
Overall survival according to the baseline level of tumor markers (P=0.027).

With a mean follow-up of 40 months, the overall 5- and 10-year survival rates were 89 and 35%, respectively. The median OS and recurrence times were 37 and 4 months, respectively. The recurrence-free survival rates were 56 and 23% at 1 and 3 years, respectively. Table II shows a univariate model of the association between clinicopathological factors and survival. The log-rank test revealed that pathological type exerted an impact on recurrence time (P=0.047; Fig. 1) and OS (P=0.048; Fig. 2). The log-rank test also revealed preoperative levels of tumor markers (P=0.027; Fig. 3) to be a prognostic factor for survival time. Treatment did not improve patient prognosis, unlike pathological type.

The multivariate analysis identified pathological type as an independent prognostic influence on survival (P=0.033; Table III). The survival characteristics were significantly different between the DPAM and PMCA-I/D or PMCA groups. The prognosis was the most favorable for DPAM. No significant difference in survival was observed with regard to treatment method (PIC or HIPEC), gender, age and the baseline level of tumor markers. Multivariate analysis of recurrence-free survival identified two significant factors: the pathological subtype (P=0.042) and the use of HIPEC (P=0.017; Table IV).

## Discussion

PMP is a low-grade malignant tumor. It is considered to be a secondary disease that originates from the appendix or ovaries (4,5). PMP generally exhibits no metastasis and rarely affects neighboring organs. The likely pathogenesis includes dissemination occurring by the rupture of appendicular adenocarcinoma or ovarian tumors with the release of neoplastic cells of mucus into the abdominal cavity. This is known as the redistribution phenomenon (6). The production of copious amounts of mucinous fluid results in a ‘jelly belly’, where the mucinous fluid accumulates in the abdomen.

PMP is a slowly progressive disease, and the pathological morphology is benign or of low-grade malignancy in the majority of cases; however, its biological behavior is malignant, and it is not able to be removed completely during surgery. Relapse may occur easily. Postoperative recurrence occurs in 60–76% of patients and the patients may have to undergo secondary surgical resections. In this study, 25 cases of PMP recurred, of which 13 cases received the second debulking surgery. The median recurrence time was four months. The recurrence-free survival rates were 56 and 23% at one and three years, respectively.

It has previously been reported that the 5- and 10-year survival rates were 50.0–81.0 and 18.2–32.0%, respectively (7). In France, a large multicentric retrospective study of 301 patients reported that the 5-year overall and disease-free survival rates were 73 and 56%, respectively (8). Furthermore, a total of 103 patients were treated at The Netherlands Cancer Institute (Amsterdam, The Netherlands), and the 3- and 5-year disease-free survival probabilities were revealed to be 43.6 and 37.4%, respectively (9). In the present study, the median OS was 37 months. With a mean follow-up of 40 months, the overall 5- and 10-year survival rates were 89 and 35%, respectively. Taking the small number of cases for the analysis into account, the statistical results may have been influenced. The 5-year survival rate was higher than that reported in the aforementioned studies.

The elevated baseline levels of CEA, AFP and CA125 were useful for diagnosing this disease. Hsieh *et al*(10) suggested that preoperatively positive CEA and CA19.9 may normalize following surgery. An increasing level of CEA and CA19–9 prognosticates early recurrence. In a study by Carmignani *et al*(11), survival was associated with preoperative CEA and CA19.9 determination. Among 532 patients, the increasing CEA level was 58% and CA19.9 level was 67.1% higher than the baseline level within one week. The elevated levels of CEA and CA19.9 were not correlated with prognosis; however, poor prognosis was associated with an elevated CEA level prior to the second surgery. Short progression-free survival was associated with the preoperative baseline CA19.9 level and favorable prognosis was associated with a normal baseline level of CA125, as shown by Baratti *et al*(12). The present study revealed that preoperative elevated baseline levels of tumor markers prolonged the survival time in univariate analyses (P=0.027). Patients with normal baseline levels of tumor markers appeared to have a more favorable survival result. The reported prognostic factors of PMP are age (13), histology, residual tumor volume (14) and intraperitoneal chemotherapy (15). Ronnett *et a1*(16) classified PMP into three histological subtypes: DPAM, PMCA and PMCA-I/D. According to the study by Ronnett *et a1*, the 5-year survival postoperatively was 75% and the 10-year survival was 68% with DPAM; this was compared with 14 and 3%, respectively, with PMCA and 50 and 21%, respectively, with PMCA-I/D. The data indicated that histological type was a significant factor for treatment and prognosis. The multivariate analysis in the present study also demonstrated that histological type was able to act as an independent prognostic factor (P=0.033). Complete CRS was able to reduce the negative prognostic impact of the pathological grade, as previously demonstrated by Elias *et al*(17). It appeared that for patients with the DPAM type, treatment supplemented by complete CRS may prolong long-term survival.

At present, it is generally accepted that aggressive CRS and HIPEC is a novel option to treat PMP. Surgical cytoreduction as a traditional treatment had a high recurrence rate, as reported by Sugarbaker (18), with a 5-year survival rate of only 20%. Furthermore, in the majority of the patients with PMP, recurrence occurred within 18 months. Repeated surgery was also likely to have increased the severity of the disease (19). According to the synergistic effect of thermochemotherapy and the different tolerances of tumors and normal tissue to temperature, Spratt *et al*(20) designed a novel treatment technique known as HIPEC. HIPEC was performed with a closed abdomen cavity for 60 min, at a temperature of 42–43ºC, using perfusate mixed with a cytotoxic drug (fluorouracil or cisplatin). The hyperthermia potentiated tumor cells on the peritoneal surface to absorb high doses of cytotoxic drugs and increased local tissue drug concentration. The aim was to eliminate microscopic and minimal residual disease remaining in the abdominal cavity following surgical resection (21). Sugarbaker (22) used the combination of CRS with PIC to treat PMP. In a cohort of 205 patients, who were classified into two groups, the 5-year survival rate was 86% for patients with HIPEC and 20% for the patients with CRS only (23). Deraco *et al*(24,25) reported a 96% survival rate at five years in 22 patients with PMP treated with HIPEC, and recurrence occurred in only one patient within a year. This demonstrated that applying HIPEC to treat PMP, in order to reduce the postoperative recurrence rate, improved quality of life and the survival rate. A large multicentric retrospective study by Elias *et al*(8) indicated that CRS combined with HIPEC should be considered as the gold standard treatment of PMP. The study underlined the prognostic impact of the extent of peritoneal seeding, particularly in patients treated by complete CRS. This prognostic impact appeared to be greater than that of the pathological grade. In our study, hyperthermic intraperitoneal chemotherapy did not have a more favorable OS rate than intraoperative intraperitoneal chemotherapy or no chemotherapy. In the present study, the recurrence time subsequent to surgery was reported from the data of the CRS and HIPEC treatment. It was suggested that this was a more accurate representation of the impact of treatment on the course of the disease. In a report from the Mayo Clinic on a subgroup analysis of 31 patients with incomplete surgical removal of mucus, intra-abdominal chemotherapy significantly reduced the recurrence (26). In the present study, HIPEC with CRS was correlated with recurrence-free survival, as shown by multivariate analysis (Table IV). As long as PMP recurred, the treatment of surgical debulking was repeated, as necessary, to alleviate pressure (26). However, repeated surgeries became more difficult due to progressively thickened intra-abdominal adhesions (27); therefore, a longer recurrence time following initial surgery was necessary to mitigate symptoms and improve the quality of life.

There were few clinical data to support how to prolong postoperative recurrence time. In the present study, stepwise multivariate Cox proportional-hazard regression analysis indicated that pathological classification and HIPEC were independent predictors of recurrence-free survival. However, due to the small sample size and the retrospective nature of the study, larger multicenter studies are required in the future.

In conclusion, the pathological subtype remained the dominant factor for survival. The efficacy of CRS with HIPEC was demonstrated in the treatment of PMP by prolonging the postoperative recurrence time. Patients with normal baseline levels of tumor markers appeared to have an improved survival result.

## Figures and Tables

**Table I:** Clinicopathological features of 39 cases of pseudomyxoma peritonei.

Number	Gender	Age (years)	Pathology type	Tumor marker				Treatment	Recurrence time (months)	Survival time (months)

				CEA	CA125	CA19-9	CA724			
1	M	61	DPAM	H	H	H	H	HIPEC	0	3+
2	F	69	DPAM	H	H	H	-	IC	32	37+
3	F	69	DPAM	H	-	H	H	CS	53	58+
4	M	61	DPAM	H	H	H	H	PIC	0	7+
5	M	58	DPAM	-	-	-	-	CS	0	8+
6	M	50	DPAM	H	H	H	H	HIPEC	1	16+
7	F	68	DPAM	-	-	-	-	CS	0	8+
8	M	65	DPAM	-	-	-	-	HIPEC	27	37
9	F	48	DPAM	-	H	H	-	CS	0	10+
10	F	65	DPAM	-	-	-	-	CS	0	11+
11	F	70	PMCA	-	-	-	-	CS	1	3+
12	F	74	PMCA-I/D	H	H	-	H	CS	0	2+
13	F	43	DPAM	-	H	H	H	CS	0	2+
14	F	67	DPAM	-	-	-	-	CS	55	18+
15	F	59	DPAM	-	H	H	H	CS	32	50+
16	M	62	DPAM	-	-	-	-	CS	48	13+
17	F	56	DPAM	H	H	H	-	CS	24	117
18	F	45	DPAM	H	H	-	-	HIPEC	0	30+
19	M	67	DPAM	H	H	H	-	HIPEC	15	32+
20	F	62	DPAM	H	-	-	-	HIPEC	0	31+
21	F	48	PMCA-I/D	H	-	H	H	IC	27	73+
22	F	70	DPAM	-	-	-	-	CS	6	25+
23	F	40	DPAM	H	-	-	H	HIPEC	0	25+
24	F	74	DPAM	-	-	-	-	PIC	4	27
25	M	45	PMCA	H	H	H	H	IC	4	55+
26	F	41	DPAM	-	-	-	-	CS	12	58+
27	F	76	DPAM	-	-	-	-	CS	1	144
28	F	34	DPAM	-	-	-	-	CS	10	58+
29	F	43	DPAM	-	-	H	H	PIC	0	60+
30	M	60	DPAM	-	-	-	-	PIC	0	50+
31	F	57	DPAM	-	-	-	-	CS	21	91+
32	M	43	DPAM	-	-	-	-	CS	6	105
33	M	47	DPAM	-	-	-	-	PIC	65	73+
34	F	80	DPAM	-	-	-	-	CS	36	75
35	M	67	PMCA	-	-	-	-	CS	4	90+
36	F	75	DPAM	-	H	-	-	CS	0	92
37	M	42	DPAM	H	-	H	H	PIC	24	93+
38	M	26	DPAM	-	-	-	-	CS	12	118+
39	M	70	DPAM	H	-	-	-	PIC	235	362

M, male; F, female; IC, intravenous chemotherapy; CS, cytoreductive surgery; PIC, perioperative intraperitoneal chemotherapy; HIPEC, hyperthermic intraperitoneal chemoperfusion; H, high; DPAM, disseminated peritoneal adenomucinosis; PMCA-I/D, peritoneal mucinous carcinomatosis with intermediate or discordant features; PMCA, peritoneal mucinous carcinomatosis; CEA, carcinoembryonic antigen; AFP, alpha-fetoprotein; CA125, cancer antigen 125; +, more than.

**Table II:** Univariate analysis of clinicopathological factors for survival.

			P-value	
			
Variable	Total (n)	Recurrence (n)	Recurrence-free survival	Overall survival
Gender			0.656	0.325
Male	15	11		
Female	24	14		
Age			0.268	0.155
≤61	21	12		
>61	18	13		
Pathological type			0.047	0.048
DPAM	34	20		
PMCA/PMCA-I/D	5	3		
Tumor maker (preoperative)			0.572	0.027
Elevated	20	15		
Normal	19	10		
Type of intraperitoneal chemotherapy			0.287	0.296
PIC	32	21		
HIPEC	7	3		

DPAM, disseminated peritoneal adenomucinosis; PMCA-I/D, peritoneal mucinous carcinomatosis with intermediate or discordant features; PMCA, peritoneal mucinous carcinomatosis; PIC, perioperative intraperitoneal chemotherapy; HIPEC, hyperthermic intraperitoneal chemoperfusion.

**Table III:** Multivariate analysis of prognostic factors for overall survival.

Variable	B	SE	Wald statistic	P-value	Exp (B)	95.0% CI
Gender	1.537	1.031	2.223	0.136	4.653	0.616–35.114
Age	−0.367	1.004	0.134	0.715	0.693	0.097–4.955
Pathology	2.530	1.189	4.523	0.033	12.549	1.219–129.152
Marker	−0.940	0.983	0.914	0.339	0.391	0.057–2.684
Treatment method	−9.954	491.896	0.000	0.984	0.000	0.000–0.000

B, unstandardized coefficient; SE, standard error; Exp, odds ratio; CI, confidence intervals.

**Table IV:** Multivariate analysis of prognostic factors for recurrence-free survival.

Variable	B	SE	Wald statistic	P-value	Exp (B)	95.0% CI
Gender	0.550	0.525	1.100	0.294	1.734	0.620–4.849
Age	0.597	0.479	1.556	0.212	1.817	0.711–4.640
Pathology	1.344	0.663	4.116	0.042	3.836	1.047–14.059
Marker	−0.740	0.527	1.974	0.160	0.477	0.170–1.340
Treatment method	2.452	1.029	5.675	0.017	11.610	1.544–87.276

B, unstandardized coefficient; SE, standard error; Exp, odds ratio; CI, confidence intervals.
